# Uric Acid Released from Poly (Lactic-co-Glycolic Acid) Nanoparticles Mitigates Glutamate-Induced Excitotoxicity of Spinal Cord Neurons

**DOI:** 10.1080/17590914.2026.2696824

**Published:** 2026-07-06

**Authors:** Kaavyashri Anbumani, Brandon J. Vaglio, Agam Aviv, Courtney M. Stains, Katie Lynch, Rene S. Schloss, Bonnie L. Firestein

**Affiliations:** a Department of Cell Biology and Neuroscience, Rutgers, The State University of New Jersey, Piscataway, NJ, USA; b Biomedical Engineering Program, Rutgers, The State University of New Jersey, Piscataway, NJ, USA; c Department of Pharmacology and Nutritional Sciences, University of Kentucky College of Medicine, Lexington, KY, USA; d Cell and Developmental Biology Graduate Program, Rutgers, The State University of New Jersey, Piscataway, NJ, USA; e Department of Biomedical Engineering, Rutgers, The State University of New Jersey, Piscataway, NJ, USA

**Keywords:** Glutamate-induced excitotoxicity, neuronal culture, nanoparticles, neuroprotection, spinal cord neurons, uric acid

## Abstract

Spinal cord injury (SCI) is often compounded by secondary damage caused by glutamate-induced excitotoxicity (GIE), where excessive glutamate release results in neuronal damage by overstimulation of glutamate receptors. This process leads to mitochondrial dysfunction, oxidative stress, and neuronal death. Uric acid (UA) has been identified as a potential neuroprotective molecule due to its antioxidant properties, but its limited solubility poses challenges for clinical use. To address this issue, we encapsulated UA in poly (lactic-co-glycolic acid) nanoparticles (UA-NPs) using a modified double emulsion technique to enhance stability and delivery of UA. Spinal cord cultures were subjected to GIE, followed by treatment with UA-NPs or empty nanoparticles. Neuronal survival was assessed with immunocytochemistry for the neuronal marker microtubule-associated protein 2 (MAP2), which revealed that treatment with UA-NPs resulted in significantly higher cell survival compared to cultures treated with empty nanoparticles. These findings suggest that UA-NPs provide neuroprotection to spinal cord neurons in vitro and may serve as a promising localized drug delivery system for SCI treatment, offering a targeted approach to mitigate secondary injury.

## PLAIN LANGUAGE SUMMARY

Spinal cord injuries can cause long-term damage not only from the initial injury but also from secondary injury processes that worsen the condition over time. One of these processes involves glutamate, an important neurotransmitter normally present in cells. However, when released in excess, glutamate can overstimulate nearby neurons and lead to damage and death of nerve cells. This process can harm essential cell functions, increase stress within cells, and reduce their ability to survive. Uric acid is a natural substance in the body that can help protect cells because of its antioxidant properties. However, it does not dissolve well in water-based solvents, including blood, making it difficult to use as a treatment. To overcome this problem, we packaged uric acid into tiny particles called nanoparticles, which improve its stability and delivery to cells. In this study, we tested our uric acid-loaded nanoparticles on spinal cord cells after exposing them to harmful conditions similar to those seen after injury. We found that cells treated with these nanoparticles survived better than those treated with empty particles. These results suggest that delivering uric acid using nanoparticles may help protect nerve cells after spinal cord injury. This approach could lead to more effective treatments that target and reduce further damage after the initial injury.

## GRAPHICAL ABSTRACT

**Figure:**
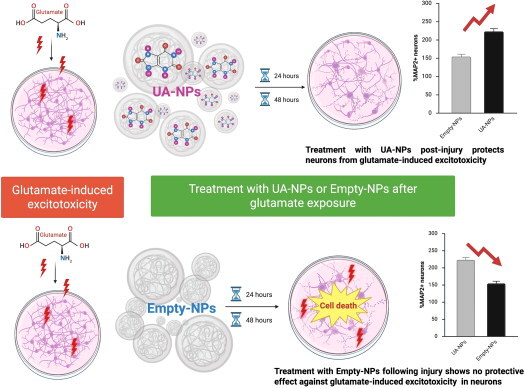

## 1. Introduction

Spinal cord injury (SCI) leads to disruptions in regular sensory, motor, and autonomic functions, with consequent effects on an individual’s physical, psychological, and social well-being (Singh et al., 2014). In the United States, there are more than 1 million individuals living with spinal cord injuries with an additional 18,000 new cases annually (Hachem et al., 2017). The primary cause of SCI is vehicular crashes, followed by falls, violence, and sports as the subsequent leading factors. Those with SCI frequently experience neuropathic pain (Singh et al., 2014), which decreases quality of life.

Treating SCI proves especially challenging as it occurs in two distinct phases. Initially, the primary phase involves cell death and tissue destruction caused by mechanical injury. In the secondary injury phase, excessive release of excitatory neurotransmitters, such as glutamate, overstimulate receptors, causing ionic imbalance. This overactivation triggers an influx of calcium ions into cells, which in turn, activates enzymes that damage cellular structures (Tian et al., 2012). Elevated calcium levels disrupt various downstream cellular processes, compounding the injury through oxidative stress and further disrupting intracellular energy metabolism, causing mitochondrial dysfunction, which results in increased production of reactive oxygen species (Torregrossa et al., 2020), ultimately causing neuronal death (Carriedo et al., 2000). Targeting this secondary phase therapeutically is optimal due to its timing in the aftermath of SCI, typically spanning hours to months after the initial injury (Torregrossa et al., 2020,Ahuja et al., 2016). Therefore, it is crucial to identify neuroprotective and regenerative therapies for enhancing clinical outcomes in SCI patients.

Uric acid is the end product of purine metabolism (Maiuolo et al., 2016) and functions as a naturally occurring antioxidant, demonstrating neuroprotective effects against SCI-induced excitotoxicity both *in vitro* (Du et al., 2007) and *in vivo* (Scott et al., 2005). Due to its antioxidant properties (Sautin & Johnson, 2008), UA is a promising therapeutic agent for SCI. Its capacity to neutralize free radicals and safeguard cells from oxidative damage constitutes a crucial mechanism underlying its neuroprotective effects (Squadrito et al., 2000). The neuroprotective properties of UA have been extensively explored in both neurodegenerative disorders and central nervous system (CNS) injuries, with studies showing that low serum UA levels are correlated with disease progression (Kutzing & Firestein, 2008). Previous studies from our group demonstrated that UA protects against glutamate-induced excitotoxicity by influencing astrocyte function, specifically by upregulating EAAT-1 glutamate transporters (Du et al., 2007). Additionally, higher plasma levels of UA correlate with better patient outcome after traumatic brain injury (Hatefi et al., 2016) and are protective in a rat model of ischemic stroke (Yu et al., 1998). These findings highlight the potential of UA as a therapy for reducing neuronal damage and promoting recovery after CNS trauma.

Administration of UA as a treatment for SCI is challenging. Although UA has strong neuroprotective effects due to its antioxidant properties, high levels of UA can lead to hyperuricemia and increases in pro-inflammatory activity (Kutzing & Firestein, 2008). This highlights the need to balance the benefits of UA as a therapeutic against potential inflammatory responses. Additionally, hyperuricemia may lead to complications, such as gout, kidney disease, and hypertension (Kutzing & Firestein, 2008,Fang et al., 2013), underscoring careful consideration of delivery methods and doses when using UA as a therapeutic intervention for SCI.

To mitigate the risks associated with systemic increases in UA levels, we have explored local delivery methods. One of the key advantages of localized delivery systems is their ability to precisely regulate the release of UA at the injury site (Singh et al., 2021), providing controlled and sustained UA delivery to protect injured neurons from secondary damage associated with SCI. This approach not only maximizes the therapeutic potential of UA but also prevents elevated UA levels throughout the body. However, the development of localized delivery systems presents challenges, particularly with the insolubility of UA in physiological solutions (Chattaraj & Paul, 2021). This emphasizes the need for innovative drug delivery technologies, such as nanofibers or nanoparticles, which can improve the bioavailability of UA at the site of injury where it is needed (Singh et al., 2021). By overcoming solubility barriers and facilitating delivery, these systems ensure that therapeutic concentrations of UA reach the injured areas, improving neuroprotection.

Here, we use poly(lactic-co-glycolic acid) nanoparticles as a delivery system for UA administration to injured spinal cord neurons in culture. Using the modified double emulsion technique, we successfully created UA encapsulated in nanoparticles (UA-NPs). We verified that the synthesized nanoparticles release UA over the first two days in solution, followed by a second release after day 4. We also performed transmission electron microscopy and demonstrated that UA-NPs are larger than empty-NPs, with both sizes being viable for biological applications. Most importantly, we demonstrated that UA released from the nanoparticles protects cultured rat spinal cord neurons from glutamate-induced excitotoxicity in an *in vitro* model of secondary injury. Overall, these data show that our UA-NPs are effective for sustained and localized UA delivery to spinal cord neurons to promote recovery after injury.

## 2. Materials and Methods

### 2.1. Preparation of UA-Loaded Poly(Lactic-co-Glycolic Acid) Nanoparticles (UA-NPs)

Uric acid (Squadrito et al., 2000) was loaded into poly(lactic-co-glycolic acid) (PLGA) nanoparticles (NPs) following a modified double emulsion technique (Perez et al., 2024,Lampe et al., 2011). UA was prepared at a concentration of 100 µg/mL (approximately 595 µM, based on a molecular weight of 168.11 g/mol) in Locke’s buffer. Briefly, PLGA (Thermofisher, cat#436200010) was dissolved in dichloromethane, and subsequently, the UA solution in Locke’s buffer was added. The emulsion was formed by placing the solution on ice and then sonicated (SFX150 Sonifier^®^, Branson Ultrasonics) for 5 seconds followed by a 30 second incubation on ice, repeated for four cycles. The emulsion was added dropwise to a 1% (w/v) polyvinyl alcohol (PVA) solution, followed by sonication as above. The resulting emulsion was added dropwise to a solution of 0.1% (w/v) PVA and placed on a rotary evaporator (Rotavapor^®^ R-300, BÜCHI) for 2 hours at 45 °C with constant rotation at 150 rpm. The particles were centrifuged for 10 minutes at 15,000 x *g*, and the supernatant was aspirated, with the NPs pelleted. Finally, the NPs were resuspended in a 2% (w/v) sucrose solution, lyophilized for 48 hours, and stored at −80 °C for subsequent analysis and future studies. Control nanoparticles were synthesized as above, excluding the addition of UA. The aspirated supernatants were saved for further analysis of loading efficiency.

### 2.2. Calculation of Loading Efficiency of UA into Nanoparticles

Encapsulation efficiency and UA loading were assessed by determining the UA concentration remaining in the supernatant from the preparation of PLGA nanoparticles. UA concentration was determined with the Uric Acid/Uricase Assay Kit (Invitrogen, cat# A22181) according to the manufacturer’s protocol. Samples were placed into a 96-well plate, and reaction mix (100 µM Amplex Red reagent, 0.4 U/ml HRP, and 0.4 U/ml uricase) was added to each well. The reaction was incubated for 30 minutes at room temperature in the dark. Absorbance was then measured with an excitation range of 530–560 nm and emission detection at ∼560 nm.

Loading efficiency percentage was determined by using the following equation:

$$\text{Loading efficiency }=\;\left(1\;-\;\left({\left[\text{UA}\right]}_{\text{remaining}}/{\left[\text{UA}\right]}_{\text{initial}}\right)\right)\times 100\%$$

### 2.3. Transmission Electron Microscopy of UA-NPs

Transmission electron microscopy (TEM) was used to characterize the size of the nanoparticles. Approximately 35 mg of UA-NPs or empty-NPs was dissolved in deionized water (diH_2_O) until the solutions became slightly cloudy. The solutions were then further diluted to a concentration of 1 mg/ml and sonicated for 1 second pulse followed by a 2 second incubation on ice repeated for 1 minute. Using negative action tweezers to suspend the EM grid in the air, 10 µL of sample was added to the grid and allowed to sit for 45 minutes. Subsequently, 5 µL Uranyless EM stain (Electron Microscopy Services, cat#22409) was added to the sample and incubated for 5 minutes. The grid containing the samples was rinsed once with diH_2_O and stored for imaging. Imaging was conducted with a Philips CM12 electron microscope.

### 2.4. UA Stability Assay

UA stock solutions (2 mM) were prepared in Locke’s buffer (NaCl, 154 mM; KCl, 5.6 mM; CaCl_2_, 2.3 mM; MgCl_2_, 1.0 mM; NaHCO_3_, 3.6 mM; glucose, 5 mM; HEPES, 5 mM; pH 7.2) and subsequently diluted to final concentrations of 50 µM, 100 µM, and 200 µM in 10 mL of 100 mM Tris–HCl pH 7.5, 100 mM NaCl. Samples were incubated at 37 °C for up to 7 days. 500 µL aliquots were collected daily for a week following resuspension for analysis. UA concentrations were determined using the Amplex Red Uric Acid/Uricase Assay Kit (Invitrogen, cat# A22181) following the manufacturer’s protocol. The assay was performed in a 96-well plate, where the reaction mix (100 µM Amplex Red reagent, 0.4 U/mL HRP, and 0.4 U/mL uricase) was added to each well. The reaction was incubated for 30 minutes at room temperature in the dark. Fluorescence was measured with excitation at 530–560 nm and emission detection at ∼560 nm. Uric acid stability data were entered into MATLAB to model uric acid degradation kinetics.

### 2.5. Determination of Release Profile of UA-NPs

NPs (1.8 or 3.6 mg/mL) were incubated in 100 mM Tris–HCl pH 7.5, 100 mM NaCl at 37 °C for a period of up to 7 days. Aliquots (500 µl) were collected daily for a week after resuspension, and UA concentrations were determined using the Amplex Red Uric Acid/Uricase Assay Kit (Invitrogen, cat# A22181) following the manufacturer’s protocol. In a 96-well plate, reaction mix (100 µM Amplex Red reagent, 0.4 U/ml HRP and 0.4 U/ml uricase) was added to each well, and the reaction was incubated for 30 minutes at room temperature in the dark. Absorbance was measured with excitation in the range of 530–560 nm and emission detection at ∼560 nm. UA concentration data were reported as concentration (µM) at each timepoint (d) to visualize whether UA levels were consistent or fluctuated when taking uric acid degradation and release from the nanoparticles into account. The empirical Amplex Red Assay data were entered into MATLAB to generate a model to predict UA concentration as a function of nanoparticle concentration and time. UA dissolved in 100 mM Tris–HCl pH 7.5, 100 mM NaCl was measured for stability and modeled by two-piecewise linear degradation functions (days 0–2 and days 2–7). To best model UA concentrations of release from the UA-NPs into 100 mM Tris–HCl pH 7.5, 100 mM NaCl and subsequent UA degradation in MATLAB, we used the same piecewise function threshold and added a model component to account for UA release from the nanoparticles. Several models were tested to fit the UA concentration data, with root mean squared error, adjusted R^2^, and Akaike Information Criterion (AIC) being used to determine the optimal fit of tested models. With the generated model, MATLAB was used to predict the UA concentration in the buffer over one week for different loading concentrations of nanoparticles between 1.8 and 3.6 mg/mL.

### 2.6. Spinal Cord Cultures

All animal procedures have been approved by the Rutgers Institutional Animal Care and Use Committee (IACUC) and are in compliance with the US Department of Health, Human Services Guide for the Care and Use of Laboratory Animals. Spinal cord tissue was isolated via Caesarian section from Sprague Dawley rat embryos at gestational day 16 (E16). The entire spinal cord was collected from each embryo, after which the meninges were removed. The collected spinal cord tissues were mechanically dissociated by gentle pipette trituration. Cells were seeded at a density of 350,000 cells/well on 24 well plates coated with 0.1 mg/ml poly-D-lysine (PDL). Spinal cord cultures were grown in serum-containing medium (SCM; 89.4% Minimum Essential Medium, 10% horse serum, 0.6% glucose) for 10 days at 37 °C and 5% CO_2_ prior to treatment. A full medium change was performed every 3 days.

### 2.7. Drug Treatments

Glutamate and UA were dissolved in Locke’s and diluted as indicated in experiments. Locke’s buffer without drugs served as vehicle.

### 2.8. Glutamate-Induced Excitotoxicity

On day *in vitro* (DIV) 10, spinal cord cultures were injured with glutamate (50 µM; L-glutamic acid) dissolved in Locke’s Buffer for 1 hour. After injury, the glutamate solution was removed and replaced with recovery medium (1:1 mixture of conditioned SCM saved before injury and fresh SCM), and cultures were incubated at 37 °C and 5% CO_2_ for 24 hours before fixation with 4% paraformaldehyde in phosphate-buffered saline (PFA). For dose-response experiments, cultures were incubated with 0 µM, 50 µM, 100 µM, 125 µM, 250 µM, 500 µM, or 750 µM glutamate.

### 2.9. Treatment with UA

On DIV 10, spinal cord cultures were treated with different concentrations of UA dissolved in Locke’s buffer for 1 hour. After treatment, the UA solution was removed and replaced with recovery medium, and cultures were incubated at 37 °C and 5% CO_2_ for 24 hours before fixation with PFA. For dose-response experiments, cultures were incubated with 0 µM, 5 µM, 10 µM, 50 µM, 100 µM, or 500 µM UA.

### 2.10. Neuroprotection Studies

Two different conditions were tested to investigate the neuroprotective effects of UA against glutamate-induced toxicity. On DIV 10, neurons were treated with glutamate (50 µM) in Locke’s buffer, containing vehicle or UA (200 µM), for 1 hour at 37 °C. Control cultures were incubated with Locke’s buffer only. After the initial 1 hour treatment, the medium was replaced with a 1:1 ratio of conditioned MEM medium and fresh medium, followed by an additional 24 hour incubation. In another set of experiments, on DIV 10, neurons were injured with glutamate as above, followed by replacement of medium containing vehicle or UA (200 µM) in a 1:1 ratio of conditioned MEM medium and fresh medium for an additional 24-hour incubation. Control cultures received 1:1 SCM medium only. Post-treatment, cells were fixed with PFA and immunostained for MAP2 (anti-MAP2, 1:1000; Millipore Sigma, cat# AB5622) to evaluate neuronal survival.

### 2.11. Treatment of Cultures with UA-NPs

On DIV10, cultures were treated with 50 μM glutamate dissolved in Locke’s buffer or with vehicle for 1 hour. Approximately 35 mg of UA-loaded nanoparticles (UA-NPs) or empty nanoparticles (empty-NPs) were dissolved in a 1:1 mixture of conditioned MEM medium and fresh MEM medium to achieve a final nanoparticle concentration of 3.5 mg/mL. For control cultures, vehicle was added for 1 hour, while treated cultures were incubated with glutamate (50 µM) for 1 hour. Post-treatment, control medium was replaced with conditioned MEM medium with or without empty-NPs or UA-NPs. Medium from glutamate-treated cultures was replaced with empty-NPs or UA-NPs in 1:1 conditioned/fresh MEM medium, and all cultures were incubated for 24–48 hours as indicated for each experiment. After the treatment period, the cultures were fixed in PFA and immunostained for MAP2.

### 2.12. Immunocytochemistry

Spinal cord neurons were grown for 10 days in culture on glass coverslips coated with 0.1 mg/ml poly-D-lysine and treated as described above for each experiment. Cultures were fixed in PFA for 10 min, permeabilized with 0.1% Triton X-100 + 5% normal goat serum in phosphate-buffered saline, and immunostained with mouse anti-MAP2 followed by secondary antibody conjugated to Alexa-Fluor^®^ 647 (1:1000; Invitrogen, cat# A-21245) or Alexa-Fluor^®^ 488 (1:1000; Invitrogen, cat# A-21245). Nuclei were stained with Hoechst 33225 dye (1:1000). After immunostaining, coverslips were mounted onto glass slides using Fluoromount-G (Thermofisher; Cat# 00-4958-02) and then visualized by immunofluorescence under a 10X objective on an EVOS M5000 microscope.

### 2.13. Imaging and Analysis

Ten images of each coverslip were randomly taken using the EVOS M5000 Cell Imaging System. Neurons immunolabeled for MAP2 were manually counted using NIH ImageJ analysis software, and the number of viable neurons per well was compared between conditions. All analyses were performed with the experimenter blinded to the condition. GraphPad Prism 10 was used to perform statistical analysis, including repeated measures ANOVA and appropriate multiple comparisons tests.

## 3. Results

### 3.1. Treatment with Glutamate Results in Loss of MAP2^+^ Neurons in Spinal Cord Cultures

**Figure 1.**
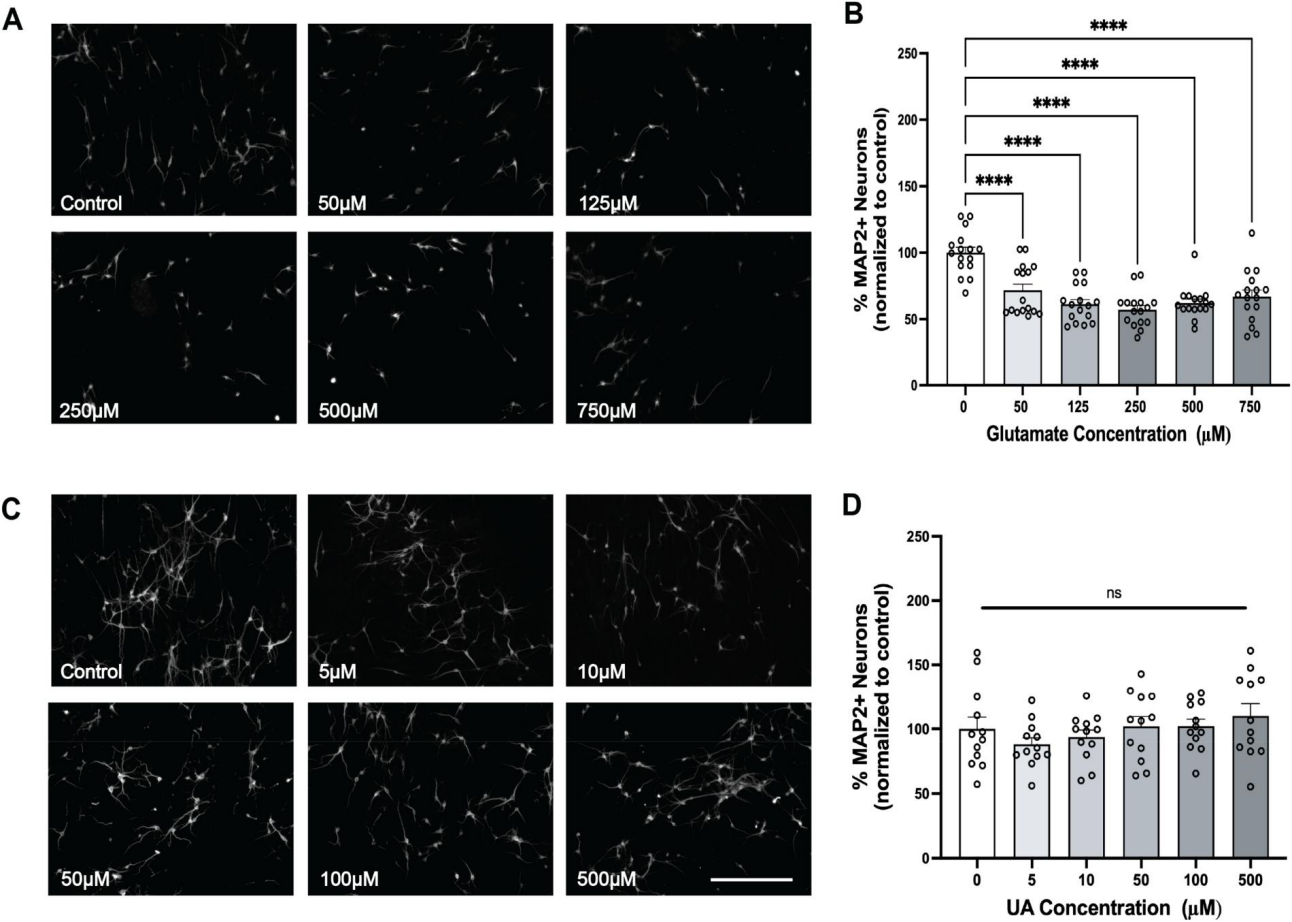
Treatment of spinal cord cultures with glutamate concentrations ≥ 50 μM result in loss of neurons. (A) Representative images of spinal cord neurons grown in SCM for 10 days (DIV10) before being injured with various concentrations of glutamate for 1 hour. Cells were fixed after 24 hours and immunostained for MAP2, a neuronal marker. (B) Quantitation of the number of surviving neurons demonstrates that exposure to glutamate concentrations between 50 μM and 750 μM results in a significant loss of spinal cord neurons. Results are from 16 wells of neurons derived from three independent experiments. **** *p* < 0.0001 as determined by one-way ANOVA followed by Dunnett’s multiple comparisons test for glutamate-treated groups versus control. Scale bar = 300 μm. (C) Representative images of spinal cord neurons on DIV 10 grown in SCM and treated with UA for 1 hour before fixation and MAP2 immunostaining. (D) UA does not affect neuronal survival as determined by MAP2 immunostaining. Results are from 12 wells derived from three independent experiments. Not significant (ns) as determined by one-way ANOVA followed by Dunnett’s multiple comparisons of UA-treated groups versus control. Data represent mean ± standard error of the mean. Scale bar = 300 μm. UA = uric acid.

Excessive glutamate released from neurons and astrocytes causes cell death following spinal cord injury (Du et al., 2007). To mimic secondary injury in SCI, we performed a dose-response study of glutamate-induced injury in spinal cord cultures. Cultures were injured for 1 hour with varying concentrations of glutamate to simulate the temporary increase in glutamate levels after SCI. We assessed neuron survival at 24 hours post-injury by counting MAP2^+^ neurons. We observed decreased neuronal survival at all glutamate concentrations compared to the uninjured control group (Figure 1A, B). MAP2-immunostaining revealed that surviving neurons maintained multiple processes and intact membrane structures (Figure 1A). Based on these findings and our previous study (Du et al., 2007), we selected a concentration of 50 μM glutamate for subsequent experiments.

### 3.2. Treatment with UA Does Not Affect Spinal Cord Neuron Survival

To determine the potential cytotoxic effect of UA on spinal cord cultures, neuronal cultures were treated with varying concentrations of UA for 1 hour. Neuron survival was assessed at 24 hours post-treatment. We found that UA concentrations up to 500 µM did not affect the number of MAP2^+^ spinal cord neurons (Figure 1C, D). Based on these findings, we selected concentrations of 100 μM and 200 μM UA for subsequent neuroprotection experiments, as these doses fall within the non-toxic range and allow for assessment of potential dose-dependent effects. These concentrations are consistent with those used in our previous studies (Du et al., 2007,Singh et al., 2021) and are physiologically relevant, as UA levels in normal human serum are reported to range from approximately 200 to 350 μM (Scott et al., 2005).

### 3.3. UA Attenuates Glutamate-Induced Toxicity in Spinal Cord Cultures

**Figure 2.**
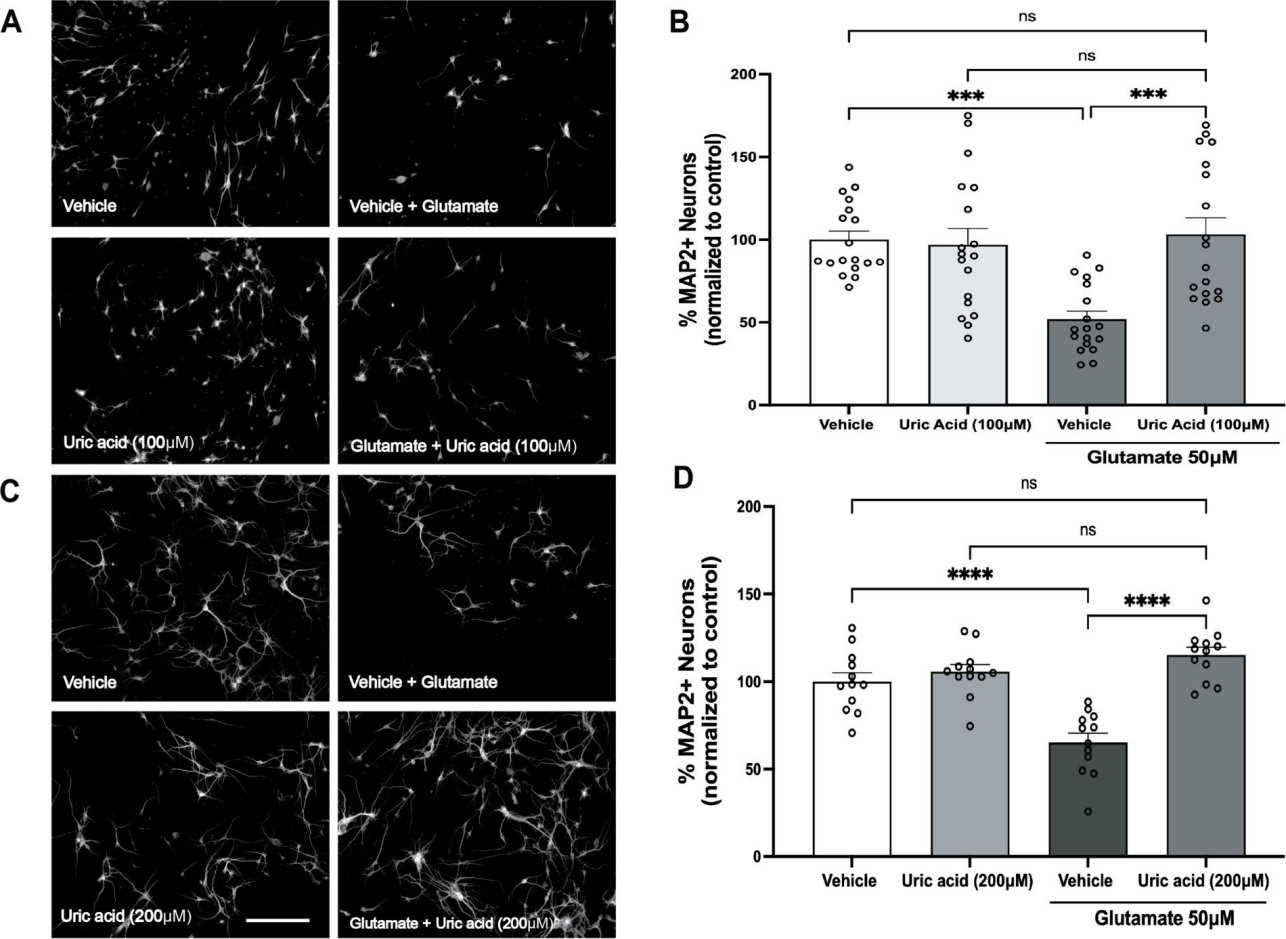
Uric acid attenuates glutamate-induced toxicity in cultured spinal cord neurons. (A) Representative images of spinal cord neurons grown in SCM for 10 days prior to treatment with 50 μM glutamate for 1 hour, with or without UA. Cells were fixed after 24 hours and immunostained for MAP2, a neuronal marker. (B) When glutamate and UA were added together, UA attenuated glutamate-induced toxicity. Results are from 18 wells from two independent experiments. *p < 0.05, **p < 0.01, ***p < 0.001, ****p < 0.0001 as determined by one-way ANOVA followed by Tukey’s multiple comparisons test using vehicle as control. (C) Representative images of spinal cord neurons cultures on DIV 10 grown in SCM and treated with glutamate for 1 hour with UA added after glutamate treatment and immunostained for MAP2. (D) When UA was added after glutamate-induced injury, neurons were protected. Results are from 12 wells from two independent experiments. ***p < 0.001, ****p < 0.0001 as determined by one-way ANOVA followed by Tukey’s multiple comparisons test using vehicle as control. Data represent mean ± standard error of the mean. Scale bar = 300 μm. UA = uric acid.

To investigate the neuroprotective effects of UA against glutamate-induced toxicity, two different experimental conditions were tested. In the first set of experiments, neurons were simultaneously treated with glutamate and either vehicle or UA in Locke’s buffer for 1 hour. Control cultures were incubated with Locke’s buffer only. After the initial treatment, the medium was replaced with a recovery medium, and cultures were incubated for an additional 24 hours. In the second set of experiments, neurons were initially exposed to glutamate for 1 hour, followed by replacement with recovery medium or recovery medium containing UA for an additional 24 hours. We observed that simultaneous treatment of cultures with glutamate and UA significantly attenuated glutamate-induced neuronal cell death (Figure 2A, B). Notably, UA treatment administered solely after the termination of glutamate exposure resulted in similar neuroprotection (Figure 2C, D). These results suggest that UA protects spinal cord neurons from glutamate-induced excitotoxicity when given after injury, which is physiologically relevant and supports its potential use in mitigating secondary injury processes following SCI.

### 3.4. Transmission Electron Microscopy Demonstrates That empty-NPs and UA-NPs Have Size Distributions That Are Consistent with Biological Applications

**Figure 3.**
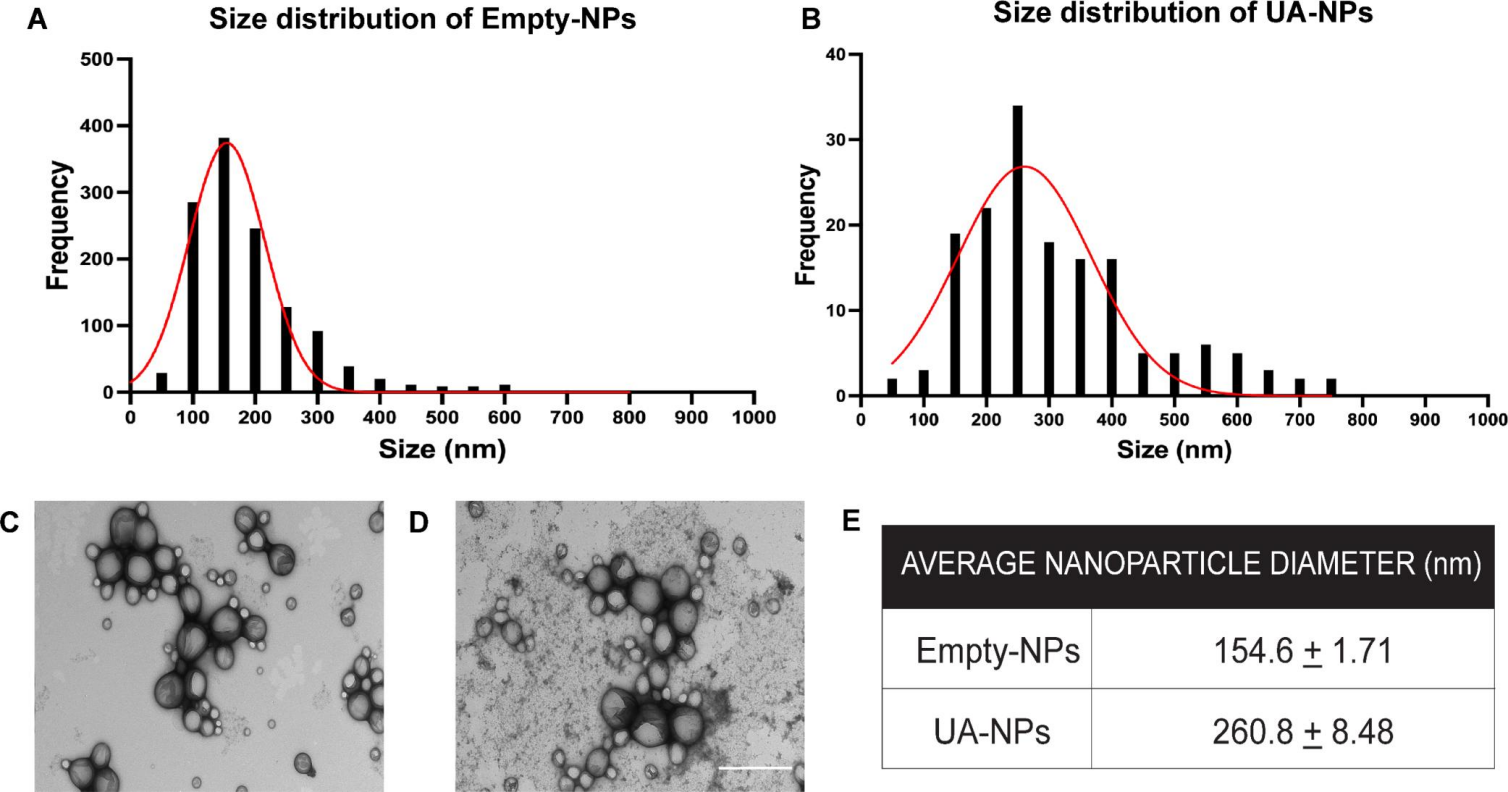
Uric acid loading increases the size of PLGA nanoparticles. (A) Size distribution of empty-NPs. (B) Size distribution of UA-NPs. (C) Representative TEM images of empty PLGA nanoparticles. (D) Representative TEM images of UA-loaded PLGA nanoparticles (E) Average diameters of empty-NPs and UA-NPs. Data represent mean ± standard error of the mean. Scale bar = 500 nm. UA = uric acid; NP = nanoparticle.

It is possible that the loading of NPs with UA results in larger NPs. To determine whether this occurs, we performed transmission electron microscopy (TEM) and characterized the size distribution of the nanoparticles. TEM analysis revealed that UA-NPs exhibited larger diameter sizes when compared to empty-NPs, with UA-NPs having an average diameter of 260.8 nm ± 8.48 nm, and empty-NPs having an average diameter of 154.6 nm ± 1.71 nm (mean + standard error of the mean) (Figure 3E). These data suggest that although the loading of nanoparticles with UA increases the nanoparticle size, both UA-loaded and empty nanoparticles are within the optimal size range for biomedical applications (Hernández-Giottonini et al., 2020,Cunha et al., 2021).

### 3.5. UA is Loaded Efficiently into PLGA Nanoparticles

The loading efficiency of uric acid (UA) into PLGA nanoparticles was evaluated by measuring the concentration of unencapsulated UA remaining in the supernatant using the Amplex Red assay. An initial UA concentration of 100 µg/mL (approximately 595 µM, based on a molecular weight of 168.11 g/mol) was used to load the nanoparticles. The PLGA nanoparticles demonstrated a high loading efficiency for UA, with an average value of 88.19% ± 0.14 (mean ± SEM). There was strong reproducibility and minimal variability across replicates, suggesting consistent nanoparticle formulation and UA encapsulation. Overall, these results confirm that the nanoparticle preparation method is highly effective for encapsulation of UA, with the majority of the initial UA successfully incorporated into the PLGA nanoparticles.

### 3.6. UA Degrades over 7 Days

We evaluated the degradation kinetics of UA at 37 °C using three different starting concentrations: 50 µM, 100 µM, and 200 µM. UA stability was measured over time using the Amplex Red assay (Supplementary Figure 1A). Percent degradation for uric acid was 38.22% ± 3.89%, 50.77% ± 3.62%, and 51.49% ± 3.14% for 50 µM, 100 µM, and 200 µM, respectively (mean ± standard error of the mean). These findings suggest that UA maintains its integrity under physiological conditions, ensuring a sustained therapeutic dose at the injury site. MATLAB modeling indicates that UA degradation fits with a first-order degradation model, whereas the rate of degradation is proportional to the concentration of UA. A two-piecewise function (days 0–2 and days 2–7) best captured uric acid stability (Supplementary Figure 1B), resulting in the following equation:

(1) C (t,C0)={0.6231 ∙ C0∙e(−0.1123 ∙ t) 0≤t≤20.4978 ∙ C0−0.1746 ∙ e(0.9531 ∙ (t−2)) 2<t≤7 

where *t* is the time (days), *C_0_* is the initial loaded uric acid concentration (µM), and *C* is the uric acid concentration (µM).

### 3.7. Release of Resuspended UA-NPs Shows Distinct Phases Corresponding to Time-Dependent Release

**Figure 4.**
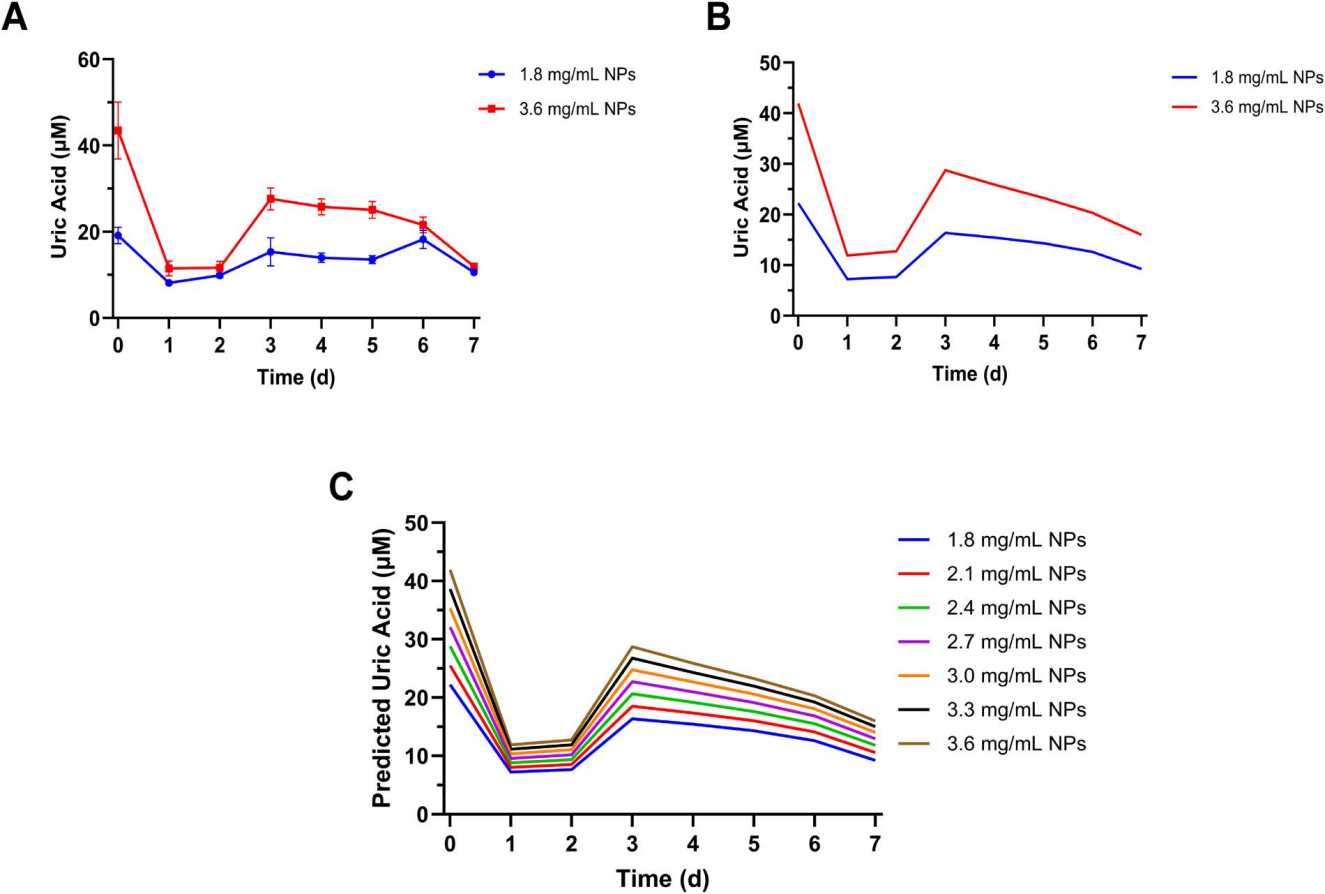
Uric acid release from PLGA nanoparticles is biphasic, with an initial release upon nanoparticle resuspension and a later sustained release. (A) Uric acid-loaded PLGA nanoparticles (1.8 and 3.6 mg/mL) show a consistent, concentration-dependent pattern of release at 37 °C in 100 mM Tris–HCl pH 7.5, 100 mM NaCl (n = 3). (B) MATLAB modeling of uric acid concentration as two-piecewise functions (days 0–2 and days 2–7) with an R^2^ = 0.93 and R^2^ = 0.76 for the two piecewise components, respectively. UA degradation is modeled with first order kinetics based on previous UA stability data, whereas UA release from the nanoparticles is measured by a second order polynomial release in piecewise function one and first order kinetic release in piecewise function two. (C) Predicted UA concentration for varying concentrations of PLGA nanoparticles between 1.8 and 3.6 mg/mL. Data represent mean ± standard error of the mean. UA = uric acid; NP = nanoparticle.

To evaluate the release kinetics of UA from PLGA nanoparticles, we incubated 1.8 mg/mL or 3.6 mg/mL nanoparticles in 100 mM Tris–HCl pH 7.5, 100 mM NaCl at 37 °C, and periodically sampled the supernatant. UA concentrations were measured over time, and the amount released, rather than cumulative release, was graphed. The Amplex Red assay results showed a similar trend for both concentrations of nanoparticles. An initial burst release of UA from UA-NPs was detected at t = 0, followed by 24 hours of UA degradation that greatly exceeded the release rate, which also occurred between days 1 and 3 (Figure 4A). Several release equations were incorporated into a piecewise model to determine UA concentration. The model test statistics (Supplementary Table 1) indicated that a second order polynomial release equation combined with the first order UA degradation equation best captured the initial phase of the piecewise function (RMSE = 3.423, R^2^ = 0.929, AIC = 98.86), whereas a first order release equation combined with a first order degradation function best models the second piecewise component (RMSE = 3.023, R^2^ = 0.763, AIC =154.342). The resulting piecewise function models the general shape of the empirical UA data (Figure 4B), and could be captured by the following equation:

(2) C(t,Cnp)={4.224 ∙ Cnp ∙ t2−11.40 ∙ Cnp ∙ t+2.570+10.93 ∙ Cnp ∙ e(−0.1123 ∙ t) 0<t≤2(2.827 ∙ Cnp+2.570)+5.395 ∙ Cnp ∙ e(−0.05040 ∙ Cnp ∙ (t−2))−(0.03900) ∙ e(0.9531 ∙ (t−2)) 2<t≤7

where *t* is the time (days), *C_np_* is the concentration of uric acid nanoparticles (mg/mL), and *C* is the uric acid concentration (µM). Several theoretical release profiles were generated by entering various UA-NP concentrations between 1.8 and 3.6 mg/mL into the model, showing the same predicted UA profile over a week (Figure 4C).

### 3.8. Treatment with UA-NPs Post-Injury Protects Neurons from Glutamate-Induced Excitotoxicity

**Figure 5.**
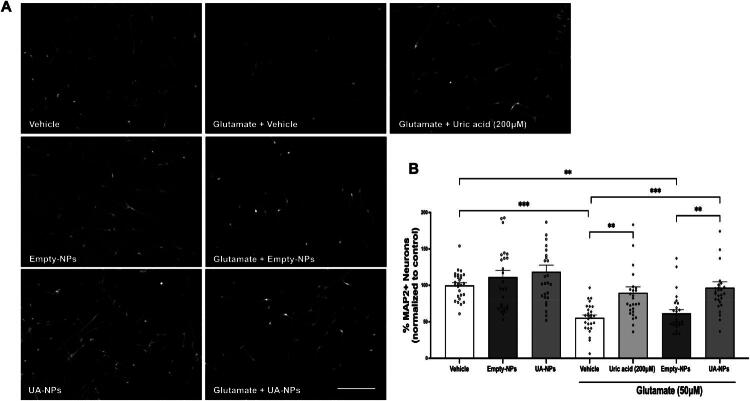
UA-NP treatment for 24 hours is neuroprotective against glutamate-induced excitotoxicity. (A) Representative images of spinal cord neurons grown in SCM for 10 days, treated with 50 μM glutamate for 1 hour, and subsequently treated with empty-NPs or UA-NPs for 24 hours. Cells were fixed after a 24 hour recovery period and immunostained for MAP2. (B) MAP2^+^ neuron counts demonstrate that treatment with UA-NPs, but not empty-NPs, is neuroprotective. Results are from 27 wells from three independent experiments **p < 0.01, ***p < 0.001 as determined by ANOVA followed by Sidak’s multiple comparisons test, compared to control. Data represent mean ± standard error of the mean. Scale bar = 300 μm. UA = uric acid; NP = nanoparticle.

**Figure 6.**
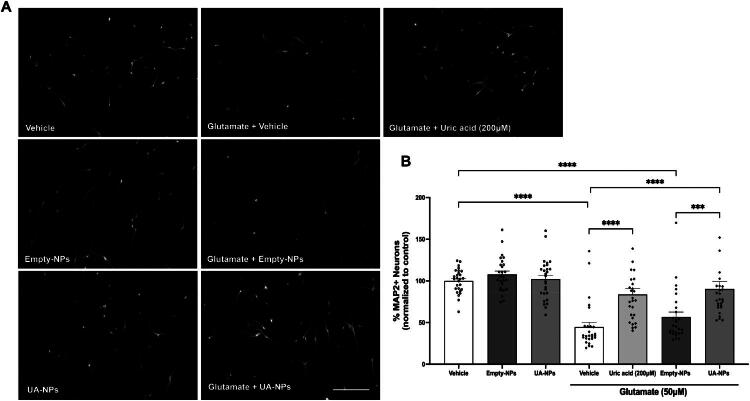
UA-NP treatment for 48 hours is neuroprotective against glutamate-induced excitotoxicity. (A) Representative images of spinal cord neurons grown in SCM for 10 days, treated with 50 μM glutamate for 1 hour, and subsequently treated with empty-NPs or UA-NPs for 24 hours. Cells were fixed after a 48-hour recovery period and immunostained for MAP2. (B) MAP2^+^ neuron counts demonstrate that treatment with UA-NPs, but not empty-NPs, is neuroprotective. Results are from 27 wells from three independent experiments. ***p < 0.001, ****p < 0.0001 as determined by ANOVA followed by Sidak’s multiple comparisons test, compared to control. Data represent mean ± standard error of the mean. Scale bar = 300 μm. UA = uric acid; NP = nanoparticle.

On DIV 10, spinal cord cultures were treated with 50 µM glutamate followed by treatment with empty-NPs or UA-NPs dissolved in recovery medium for 24 or 48 hours. MAP2^+^ neuron counts demonstrate that injured cultures treated with UA-NPs for either 24 hours (Figure 5) or 48 hours (Figure 6) exhibited significantly lower levels of neuronal death compared to those treated with empty-NPs. Additionally, treatment with empty-NPs, either in control or injured cultures, did not promote cell death. Our data suggest that the UA released from the nanoparticles facilitates neuronal protection after injury and that UA-NPs may be a viable therapeutic for SCI.

## 4. Discussion

Our current study investigated the potential of UA-loaded nanoparticles (UA-NPs) as a therapeutic delivery system for attenuating glutamate-induced excitotoxicity in cultured spinal cord neurons. Using dissociated spinal cord cultures as an *in vitro* injury model, we demonstrated that treatment with UA-NPs significantly mitigates neuronal death caused by excess glutamate. Neuronal numbers were consistently higher with treatment with UA-NPs compared to treatment with empty-NPs at both time points tested, highlighting the robust neuroprotective effects of UA-NPs in sustaining neuronal survival post-injury. These findings align with previous studies demonstrating the neuroprotective properties of soluble UA against excitotoxicity (Du et al., 2007) and complement earlier work utilizing UA-polycaprolactone (UA-PCL) fibers, which protect spinal cord tissues from similar injuries (Perez et al., 2024). Collectively, these results establish UA-NPs as a promising therapeutic candidate for treatment of SCI.

Several other small molecules have been evaluated for neuroprotection in SCI and provide context for UA-NP development. Classical agents, such as methylprednisolone and tirilazad, reduce lipid peroxidation and inflammation but are limited by systemic toxicity and inconsistent clinical benefit (Hall, 2011). More recently, ferroptosis inhibitors, including ferrostatin-1 and its soluble analog UAMC-3203, have been shown to reduce lipid peroxidation, restore glutathione metabolism, and improve functional recovery after SCI (Huang et al., 2025). Natural polyphenols, such as quercetin, also exert antioxidant and anti-inflammatory effects by activating Nrf2 signaling pathways (Shen et al., 2024), and Riluzole, a glutamate-modulating molecule with clinical relevance, reduces excitotoxicity in SCI models (Nagoshi et al., 2015). These molecules illustrate the diversity of biochemical pathways contributing to secondary SCI and underscore the need for delivery platforms that can maintain effective therapeutic levels of relevant molecules in the injured spinal cord. Furthermore, UA is a viable therapeutic candidate due to the fact that it is an exogenously produced molecule, has reported redox activity, and has demonstrated safety in human neurological trials (URICO-ICTUS Investigators, 2014). However, systemic UA delivery carries risks, including the development of gout and altered purine metabolism (Kutzing & Firestein, 2008). NP encapsulation provides the first step of a strategy for local UA delivery to reduce systemic exposure and extend the therapeutic window of UA.

Poly-lactic-co-glycolic acid (PLGA), the polymer used in this study, is widely recognized for its utility in the sustained delivery of small macromolecules (Makadia & Siegel, 2011,Bertram et al., 2010). For instance, PLGA nanoparticles have been used to deliver α-tocopherol for SCI treatment in an *in vitro* model (Laliwala et al., 2022), and PLGA microparticles loaded with melatonin have been used for treatment of SCI-induced neuropathic pain *in vivo* (Zhang et al., 2021). Similarly, encapsulation in PLGA nanoparticles protects brain-derived neurotrophic factor (BDNF) from degradation and enables sustained, controlled release while preserving bioactivity (Perez et al., 2024). This results in significantly enhanced neuronal survival *in vitro* and improved neuroprotection (Perez et al., 2024). PLGA nanoparticles coated with poloxamer 188 (PX) have been used to facilitate effective BDNF delivery, leading to improved cognitive and neurological outcomes post-TBI in mice (Khalin et al., 2016). PLGA-based biomaterials are completely absorbed by the body post-surgery and present substantial potential as carriers for local and sustained UA release. Their use as clinical suture material attests to their biocompatibility, excellent safety profile, and tunable rates of biodegradability *in vivo*, with FDA approval for drug administration (Ding & Zhu, 2018). Consequently, in our study, PLGA-based nanoparticles (NPs) were chosen as a vehicle for UA delivery, capitalizing on their hydrophobic carrier properties (Mitchell et al., 2021).

Our UA-NP release studies revealed that UA is released from PLGA nanoparticles in a biphasic pattern, including an immediate burst of release. It is possible a portion of the uric acid loosely adsorbs to the UP-NP surface and quickly releases once added to the solution (Siepmann & Siepmann, 2025). However, there is also the possibility that the varying nanoparticle size could contribute to the biphasic release. A study on Gefitinib-loaded PLGA nanoparticles examined the release kinetics, and the smallest size fraction showed an instantaneous release of Gefitinib within 5 days while the largest size fraction had not released the entirety of drug load after 90 days (Chen et al., 2017). Similar size-dependent variations in drug release between PLGA nanoparticles and microspheres were reported when measuring the release of ciprofloxacin (Hua et al., 2014) and DiI (Sahin et al., 2017). PLGA nanoparticles are reported to be stable over one week at 37 °C, with minimal erosion of the PLGA matrix (Jain et al., 2010). The observed delayed secondary release is likely attributed to the gradual diffusion of uric acid from the nanoparticles. This profile indicates that while UA can be effectively released from the nanoparticles, rapid initial release suggests the need for additional modifications, such as alginate encapsulation (Perez et al., 2024), for more sustained UA delivery at the injury site. Studies using PLGA nanoparticles as neurotherapeutics have shown mixed results on whether the nanoparticles are taken up by the neurons or flow freely. Several formulations of PLGA nanoparticles are taken up by SK-N-SH cells (Djiokeng Paka et al., 2016), but fluorescein isothiocyanate-conjugated PLGA nanoparticles injected *in vivo* showed low accumulation in spinal cord neurons compared to microglia (Choi et al., 2025). The size of nanoparticles impacts the efficiency in which nanoparticles accumulate in cells, with smaller nanoparticles (60 nm) being taken up more efficiently compared to larger nanoparticles (90–120 nm) (Mok, 2024,Meng et al., 2022). Due to the increased diameter of our UA-NPs, it is possible that the ability of neurons to endocytose the UA-NPs is decreased when compared to empty PLGA nanoparticles and that there is a higher concentration of free-flowing UA-NPs in the medium.

Additionally, UA stability assessments confirmed that UA degradation varies between 38.22% and 51.49% depending on the starting concentration (50 µM, 100 µM, and 200 µM) over the tested incubation period at 37 °C. These findings demonstrate some resistance of UA to degradation under physiological conditions but also points to a need to replenish UA concentrations at the injury site to extend therapeutic use and optimization of bioavailability and therapeutic effectiveness in SCI treatment. Delayed release of UA from drug carriers is one approach to ensure maintenance of UA concentration. In addition to controlling release kinetics, localized nanoparticle delivery represents an important future strategy for translating UA-NPs toward SCI therapy. Intrathecal administration is particularly relevant because it bypasses systemic circulation and provides direct access to the cerebrospinal fluid surrounding the spinal cord (Householder et al., 2019). Intrathecally administered nanoparticles have been shown to distribute throughout the central nervous system and remain associated with the leptomeninges for more than three weeks, highlighting the potential of this route for sustained local nanomedicine delivery (Householder et al., 2019). The successful intrathecal delivery of PLGA nanoparticles incorporated within hyaluronan/methylcellulose hydrogels following SCI further demonstrates the potential of biomaterial-assisted approaches for localized and sustained therapeutic delivery (Baumann et al., 2010). Therefore, future UA-NP formulations could be administered intrathecally, or incorporated into injectable hydrogels or scaffolds, to maintain therapeutic UA concentrations near the injured spinal cord while reducing systemic exposure and associated side effects.

There is currently no evidence to suggest that uric acid (UA) directly binds to glutamate. Instead, prior work, including our previous study (Du et al., 2007), supports an indirect mechanism of protection. This prior study demonstrated that UA does not protect neurons in pure neuronal cultures but only demonstrates a protective effect when astroglia are present, indicating that its neuroprotective action is astrocyte-dependent rather than due to direct interaction with glutamate. Importantly, this work also suggests that UA does not act solely by scavenging toxic byproducts, such as peroxynitrite, but instead plays an active role in modulating glial function. Specifically, UA enhances uptake of glutamate by astrocytes via upregulation of EAAT-1 glutamate transporters, reducing excitotoxic stress. Taken together, these findings indicate that UA attenuates glutamate-induced toxicity by acting on astroglia, reducing downstream consequences of excitotoxicity rather than by binding glutamate.

## 5. Conclusion

In summary, our study underscores the promise of UA-NPs as a novel therapeutic approach for SCI. The biocompatibility, safety, and tunable properties of PLGA nanoparticles provide a robust platform for UA delivery, enabling effective reduction of neuronal death induced by GIE. The significant neuroprotection of spinal cord neurons observed with UA-NP treatment supports UA-NPs as a viable intervention for SCI. These findings contribute to a growing body of evidence that UA has a neuroprotective role post-injury and emphasize the importance of innovative biomaterials for SCI treatment. Future directions will focus on optimizing nanoparticle formulations and translating these findings to clinically-relevant models to address the unmet therapeutic needs of patients with SCI.

## 6. Supporting Information

Time-dependent degradation of uric acid over one week in vitro that can be modeled using first-order kinetics (Supplementary Figure 1); metrics used to quantify the different fits of model to the uric acid degradation and release data from nanoparticles (Supplementary Table 1).

## Supplementary Material

Supplemental Material

## Data Availability

The data that support the findings of this study are available from the corresponding author upon reasonable request.

## Abbreviations

DIV: days *in vitro*
GIE: glutamate-induced excitoxicity
NPs: nanoparticles
PDL: poly-D-lysine
SCI: spinal cord injury
UA: uric acid
UA-NPs: uric acid-loaded nanoparticles.
