# Supplementary material for: Uric Acid Released from Poly (Lactic-co-Glycolic Acid) Nanoparticles Mitigates Glutamate-Induced Excitotoxicity of Spinal Cord Neurons
Source: ASN Neuro. 2026 Jul 6;18(1):2696824. doi: 10.1080/17590914.2026.2696824 (PMC13349032; doi:10.1080/17590914.2026.2696824)
Supplement: Supplemental Material [file TASN_A_2696824_SM6977.docx]

SUPPLEMENTARY FIGURES


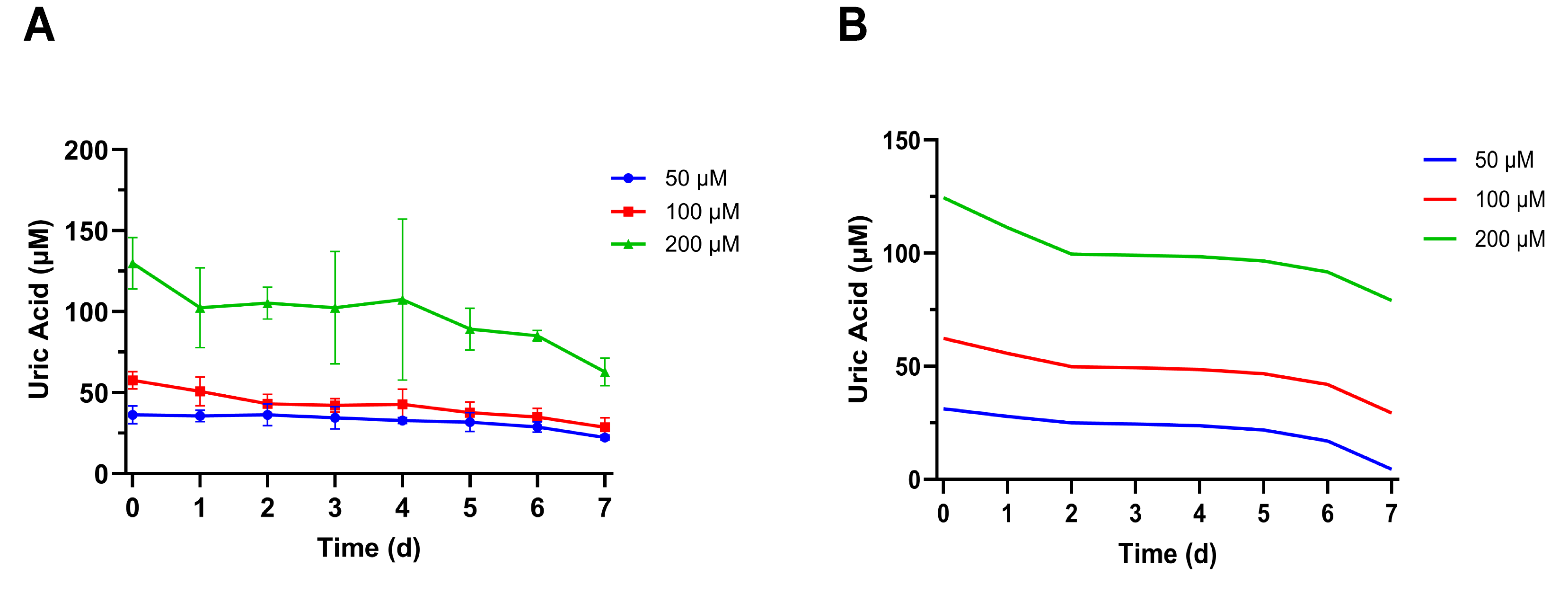


**Supplementary Figure 1. Uric acid degradation over the course of one week *in vitro* can be modeled with first order kinetic equations.** A) UA at concentrations of 50 µM, 100 µM, or 200 µM in 100 mM Tris–HCl pH 7.5, 100 mM NaCl degrade over one week at (n = 3). B) MATLAB modeled UA degradation as two-piecewise functions (days 0-2 and days 2-7) with an R^2^ = 0.89 and R^2^ = 0.71 for the two piecewise components, respectively. Data represent mean ± standard error of the mean. UA = uric acid.

| **Model Name** | **RMSE - Piecewise 1** | **RMSE - Piecewise 2** | **Adjusted R^2^ - Piecewise 1** | **Adjusted R^2^ - Piecewise 2** | **AIC - Piecewise 1** | **AIC - Piecewise 2** |
| --- | --- | --- | --- | --- | --- | --- |
| Model 1 | 3.423337741 | 3.06281583 | 0.92855116 | 0.75668419 | 98.8603043 | 155.13557846 |
| Model 2 | 8.518416373 | 5.47244200 | 0.557601738 | 0.22323277 | 131.6784238 | 190.82678390 |
| Model 3 | 6.420824626 | 3.95371177 | 0.74865092 | 0.59454881 | 121.9053686 | 170.45478610 |
| Model 4 | 4.693266581 | 8.23328541 | 0.865709241 | -0.75822681 | 108.6223308 | 214.46660459 |
| Model 5 | 6.868188178 | 8.07283731 | 0.712405845 | -0.69036682 | 122.3301049 | 212.37682636 |
| Model 6 | 8.518416373 | 3.44354623 | 0.557601738 | 0.69243253 | 131.6784238 | 162.16560589 |
| Combined Model | **3.423337741** | **3.02254721** | **0.92855116** | **0.76304016** | **98.8603043** | **154.34149193** |

**Supplementary Table 1: Test statistics for models of UA degradation and release from the PLGA nanoparticles indicated combining a second order polynomial release function with the first order degradation of uric acid best captured the uric acid concentration for piecewise component one (days 0-2), whereas a first order release function combined with the first order degradation of uric acid best captured uric acid concentration for piecewise component two (days 2-7).** UA = uric acid.
